# Diet Supplementation with Ketoanalogues, Inulin, and Calcium Citrate in Chronic Kidney Disease: A Retrospective Cohort

**DOI:** 10.3390/life14121638

**Published:** 2024-12-10

**Authors:** Martín Calderón-Juárez, Nadia Saavedra-Fuentes, Karla Guadalupe Del Castillo-Loreto, Juan Carlos Castillo-Salinas, Claudia Lerma

**Affiliations:** 1 Faculty of Sciences, Universidad Nacional Autónoma de México, Coyoacan 04510, Mexico; martincj@ciencias.unam.mx; 2 Centro Medico Dalinde, Mexico City 06760, Mexico; dra.nadiasaavedra.nefrologia@gmail.com; 3 Independent Practice, Mexico City 06760, Mexico; karla.delcastillo9@hotmail.com; 4 Independent Practice, Mexico City 03010, Mexico; juancarloscassal@gmail.com; 5 Department of Molecular Biology, Instituto Nacional de Cardiología Ignacio Chávez, Tlalpan 14080, Mexico; 6 Faculty of Health Sciences, Universidad Anahuac Mexico, Huixquilucan 52786, Mexico

**Keywords:** chronic kidney disease, ketoanalogues, retrospective cohort

## Abstract

The addition of ketoanalogues (KAs) to a low-protein diet has been shown to mitigate the progression of pre-dialysis chronic kidney disease (CKD). The addition of inulin and calcium citrate may add further benefits, given their nephroprotective effects. In this study, we tested the changes in estimated glomerular filtration rate (eGFR), CKD symptoms, body composition, and biochemical parameters after 6 months of diet supplementation with Cetolán III, a combination of KA, inulin, and calcium citrate. We included 76 adult patients diagnosed with CKD stages 3 and 4 and not treated with renal replacement therapy in a retrospective cohort. In this cohort, participants were followed through two clinic visits at 3 and 6 months after diet supplementation. We found a slight increase in eGFR at 3 and 6 months compared with baseline, as well as a decrease in the severity of CDK-related symptoms, fat mass, and muscle mass. We observed only a slight decrease in creatinine and uric acid after 6 months of follow-up. We did not find a remarkable change in anthropometric parameters (e.g., body mass index, waist circumference, and arm muscle area). This observational study suggests that addition of KA, inulin, and calcium citrate to a low protein- diet could be associated with an improvement in eGFR and symptom severity in CKD pre-dialysis.

## 1. Introduction

Chronic kidney disease (CKD) is the slow, progressive loss of kidney function over years. The goal of medical treatment is the prevention or slowing of renal deterioration [1]. Among the most common causes of CKD are type 2 diabetes mellitus, systemic arterial hypertension, lupus, and vasculitis [1]. The estimated glomerular filtration rate (eGFR) is used to classify CKD into six clinical stages (G1–2, G3a, G3b, and G4–5), this functional measurement corresponds to the volume of fluid filtered per unit of time by the kidneys [2]. Usually, stages G1–G4 are managed by medical treatment that does not involve kidney replacement treatment (such as dialysis and kidney transplant).

One of the kidneys’ main functions is excreting toxins, including uremic toxins (such as urea and creatinine) [3]. Since protein intake is the main source of uremic toxins, part of the treatment of CKD includes a change in diet, primarily focused on protein restriction [1]. However, protein restriction in the diet is associated with a high risk of malnutrition in patients with CKD.

Treating malnutrition is of paramount importance in CKD. Its prevalence is 35.2–50.6% [4], and it is related to negative outputs, such as muscle dysfunction [5], dyslipidemia, and mortality [6]. Nutritional supplementation with specific compounds is recommended to mitigate malnutrition and its consequences in CKD, including ketoanalogues (KAs), folic acid, vitamins, and other micronutrients [7]. Here, we focus on the combination of three compounds that have been shown to have a nephroprotective effect in animal models: KAs, calcium citrate, and inulin [8]. Such compounds are commercialized as an oral nutritional supplement known as Cetolán III (Cetolán Laboratorios Columbia™ Reg. No. 122M2016 SSA IV) [8].

Briefly, a protein restriction diet plus KA supplementation is recommended in patients with CKD because it has been demonstrated to improve several clinical outcomes, including eGFR, nutritional status, and patient survival [7]. Calcium-based supplements are advised to maintain a neutral balance, due to their important role in bone and muscle health, among others [7]. Inulin is a neutral-tasting prebiotic fiber that has shown to improve nutritional, cardiovascular, and psychological parameters in patients with CKD [9].

Although supplementation with KA and calcium is supported by clinical guidelines and the use of inulin has been shown to improve several biochemical and nutritional parameters in patients with CKD in some human studies [7,9,10], the biological effects of combined supplementation with KA, calcium, and inulin are unknown in humans. In addition to the improvement in eGFR and uremic levels, these nutritional supplements can potentially improve disease progression and patient-reported outcomes [9,11,12], such as CKD symptom severity. Furthermore, patients, health personnel, and researchers have urged a broadening of the research scope to patient-reported outcomes that might be relevant to their physical functioning and well-being [13].

In this work, we investigated a retrospective cohort of patients with pre-dialysis CKD who had been prescribed a low-protein diet and KA, calcium, and inulin (Cetolán III) oral supplementation, the potential change of urea, eGFR, and symptom severity after three and six months of intake.

## 2. Materials and Methods

### 2.1. Study Design and Participants

In this retrospective cohort, we consecutively obtained data from the clinical records of patients with CKD to whom Cetolán III was prescribed by their nephrologist from 2020 to 2023 at six clinics in Mexico. We included adult patients with an eGFR < 60 mL/min who were not treated with renal replacement therapy. The baseline data were obtained on the day Cetolán III was prescribed and subsequent data were obtained for the following six months. Some data and patients were lost during follow-up in months 3 and 6, and the number of patients included in the analysis for every variable is reported accordingly. We excluded patients with cancer and those who were taking other dietary supplements. All procedures were conducted following the Declaration of Helsinki. This study was approved by the Ethics Committee of Centro Médico Dalinde (protocol CINV-000001).

### 2.2. Data Gathering

The records of patients who received supplementation with Cetolán III were located. Those who had completed supplementation for at least six months were selected. Patient information was obtained from files (nutritional clinical history) stored in physical form (printed) at the clinic where the patient received their follow-up. PDF and Excel copies were obtained (eliminating patient identification data), which consisted of three files: (1) initial consultation, (2) follow-up consultation at 3 months, and (3) follow-up consultation at 6 months. Copies were sent to the main researcher for information extraction and safekeeping. Each file was reviewed by the associate researcher to determine if it met the inclusion and exclusion criteria and to send it to technical support with a note indicating whether it met the criteria to be included in the study. For any clinical or operational questions, the clinical expert (N.S.-F.) was consulted. Each file with the accompanying researcher’s notes was then reviewed to extract the information on the study variables in an electronic data collection sheet (Excel format). The senior investigator supervised the information in each file captured electronically to determine if the case had the necessary information complete.

### 2.3. Study Variables

(a) The main dependent variables of the study were serum urea levels (mg/dL), eGFR (mL/min/1.73 m^2^), and symptom severity. The independent variables were age, sex, time since CKD diagnosis, history of the most frequent relevant comorbidities (diabetes, hypertension, or autoimmune disease), edema, smoking, alcoholism, and drug consumption.
(b) The severity of symptoms was graded by patients as none, mild, moderate, and severe. The included symptoms were nausea, vomiting, pyrosis, diarrhea, abdominal distention, constipation, anorexia, pruritus, and dyspnea.
(c) Other renal function and biochemical parameters comprised serum creatinine (mg/dL), uric acid (mg/dL), glucose (mg/dL), total cholesterol (mg/dL), triglycerides (mg/dL), low-density lipid (LDL, mg/dL), high-density lipid (HDL, mg/dL), calcium (mg/dL), sodium (mEq/L), phosphate (mEq/L), and potassium (mEq/L).
(d) Body composition and arm dynamometry parameters comprised body fat (kg), body fat percentage (%), muscle mass (%), muscular strength, body mass index (kg/m^2^), waist circumference (cm), and arm muscle area (cm^2^).
(e) Other biochemical parameters and blood count parameters comprised albumin (g/dL), hemoglobin (g/dL), hematocrit (%), and iron (mcg/dL).
(f) Body composition parameters assessed by bioelectrical impedance parameters were total water (L), extracellular water (L), and phase angle (°).

### 2.4. Statistical Analysis

We used SPPS version 21.0 and considered *p*-values less than 0.05 to be statistically significant. For quantitative continuous variables with abnormal distribution based on the Kolmogorov–Smirnov test, we compared them using the Wilcoxon signed-rank test (data reported as medians (percentile 25–percentile 75)). Also, we report changes in quantitative variables at months 3 and 6 (Δ). To test the statistical significance of such values, we performed a Wilcoxon rank test. Categorical variables are reported as absolute frequency and relative frequency. For our main output, change in eGFR, we performed a post hoc analysis to test the achieved statistical power of the change (Δ) between baseline and 6-month follow-up using G*Power 3.1.9.7 software [14]. With an effect size of 0.49 and an α error probability of 0.05, we estimated an achieved statistical power (1—β error probability) of 0.88. In biomedical research, a statistical power greater than 0.8 is usually accepted to test a hypothesis [15].

## 3. Results

A total of 76 patients were included in our study, and demographic and clinical characteristics are shown in Table 1. We describe the results in three sections: symptoms, renal function and biochemical parameters, and body composition. Also, we provide highlights of some of the most relevant results.

### 3.1. Symptoms

**Figure 1:**
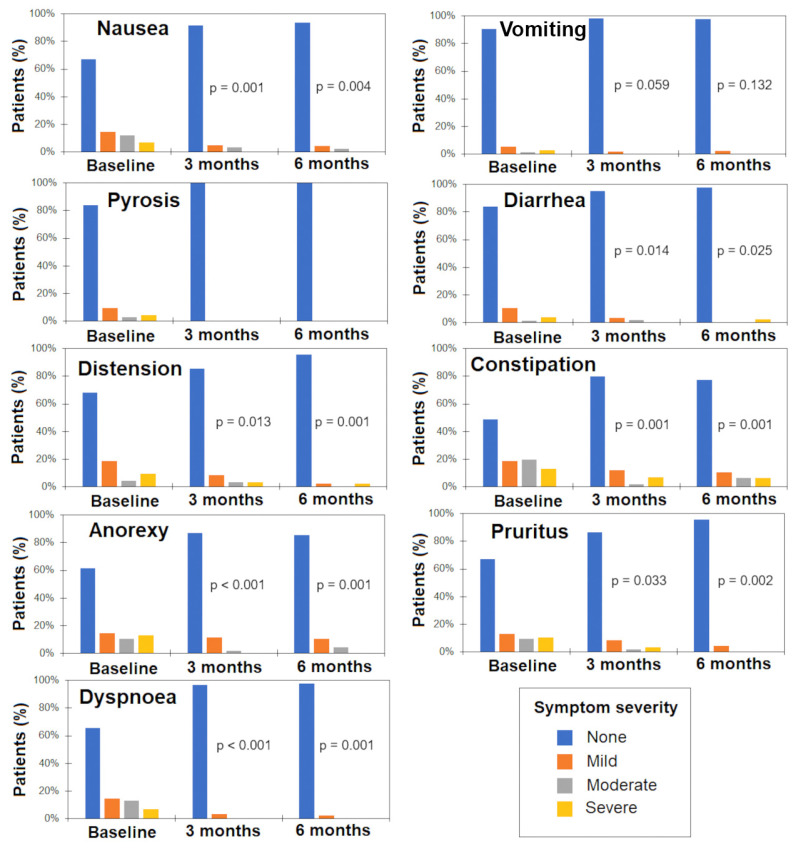
Severity of CKD-related symptoms (N = 76) at baseline and 3 and 6 months after dietary supplementation with Cetolán III.

Figure 1 shows the proportion of patients with nausea, vomit, pyrosis, diarrhea, abdominal distention, constipation, anorexia, pruritus, and dyspnea at baseline and 3 and 6 months. The severity of symptoms was graded by patients as mild, moderate, and severe. Compared with baseline, the severity of nausea, diarrhea, abdominal distention, constipation, anorexia, pruritus, and dyspnea had diminished significantly at the 3- and 6-month follow-ups.

### 3.2. Renal Function and Biochemical Parameters

**Figure 2:**
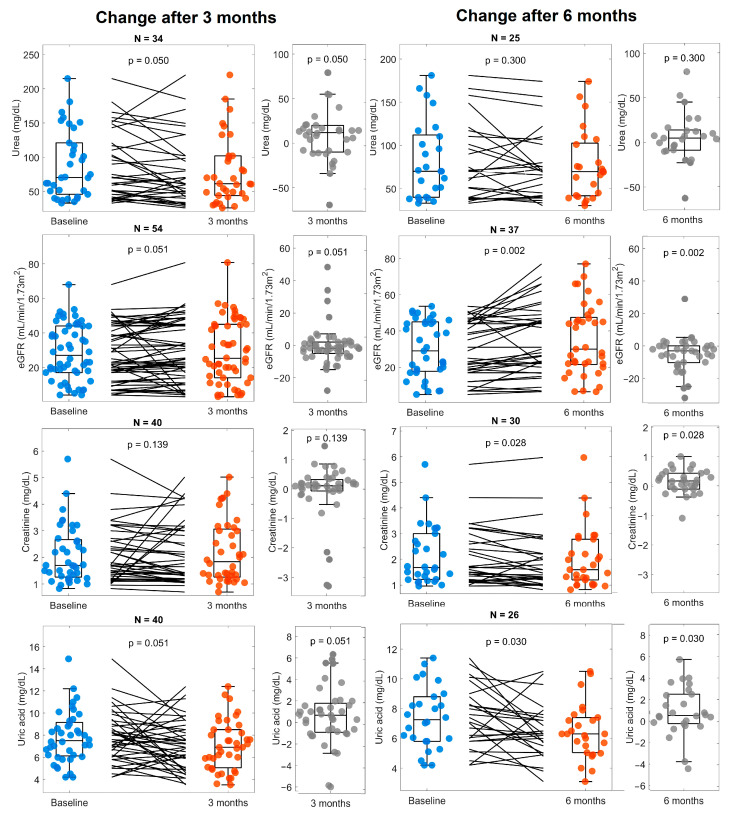
Uremic toxins and eGFR at baseline and 3 and 6 months. Panels of the left compare values at baseline (blue) compared with 3 months (orange), as well as the change between them (gray). Panels on the right show the changes observed after 6 months (orange) compared with baseline (blue) and the change between them (gray). The number of subjects included for the analysis of every variable is shown at the top of every panel.

Figure 2 shows the levels of urea, eGFR, creatinine, and uric acid during the study. We did not observe an increase in eGFR or a change in creatinine and uric acid until six months after nutritional supplementation. After six months, we found a statistically significant higher eGFR, as well as a decrease in creatine and uric acid levels.

**Figure 3:**
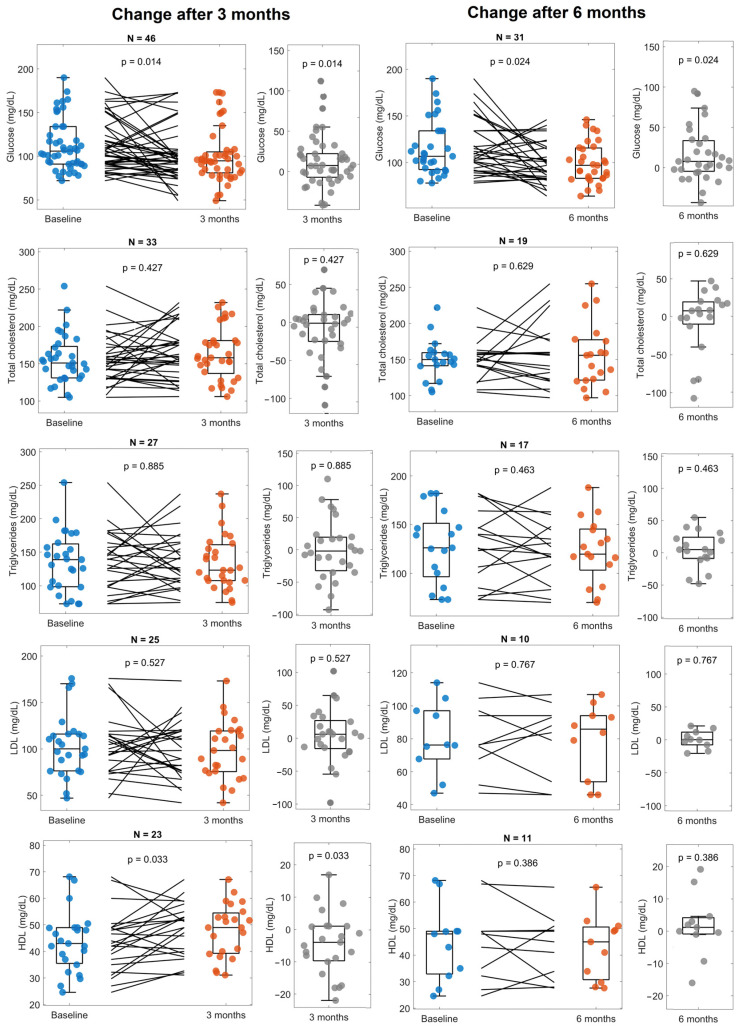
Glucose, total cholesterol, triglycerides, LDL, and HDL at baseline and 3 and 6 months. The panels on the left compare values at baseline (blue) with 3 months (orange), as well as the change between them (gray). Panels on the right show the changes observed after 6 months (orange) compared with baseline (blue) and the change between them (gray). The number of subjects included for the analysis of every variable is shown at the top of every panel.

**Figure 4:**
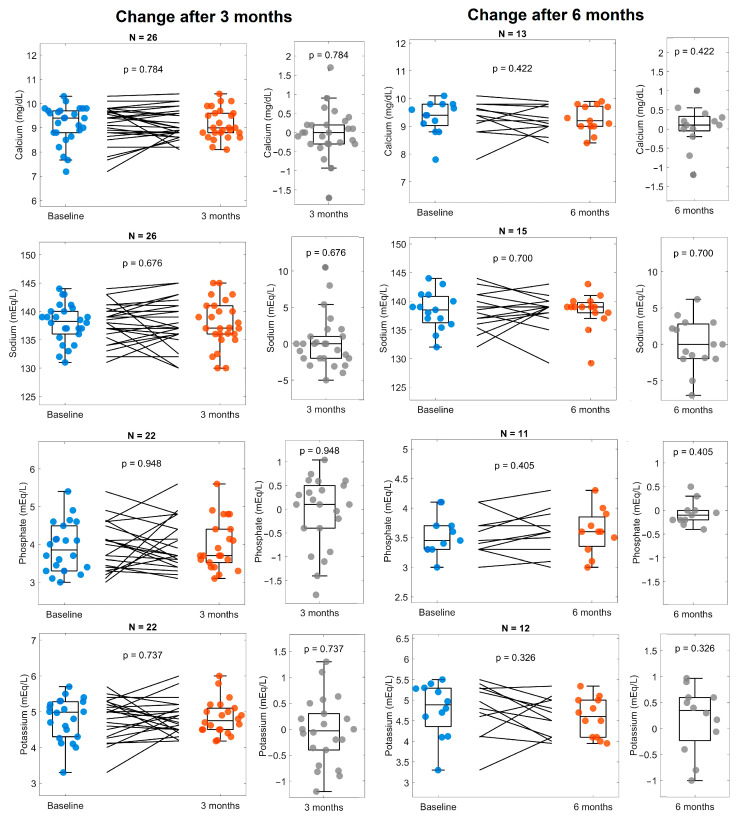
Electrolytes (calcium, sodium, phosphorus, and potassium) at baseline and 3 and 6 months. The panels on the left compare values at baseline (blue) with 3 months (orange), as well as the change between them (gray). Panels on the right show the changes observed after 6 months (orange) compared with baseline (blue) and the change between them (gray). The number of subjects included for the analysis of every variable is shown at the top of every panel.

A small decrease in glucose was observed after three months and six months (Figure 3). There were few statistically significant changes found in the lipid profile (total cholesterol, triglycerides, LDL, and HDL), except for an increase in HDL after 3 months of Cetolán III supplementation; however, this was not observed after 6 months. Furthermore, we did not find changes in electrolyte serum levels (calcium, sodium, phosphate, and potassium) at any point of the follow-up (Figure 4).

### 3.3. Body Composition

**Figure 5:**
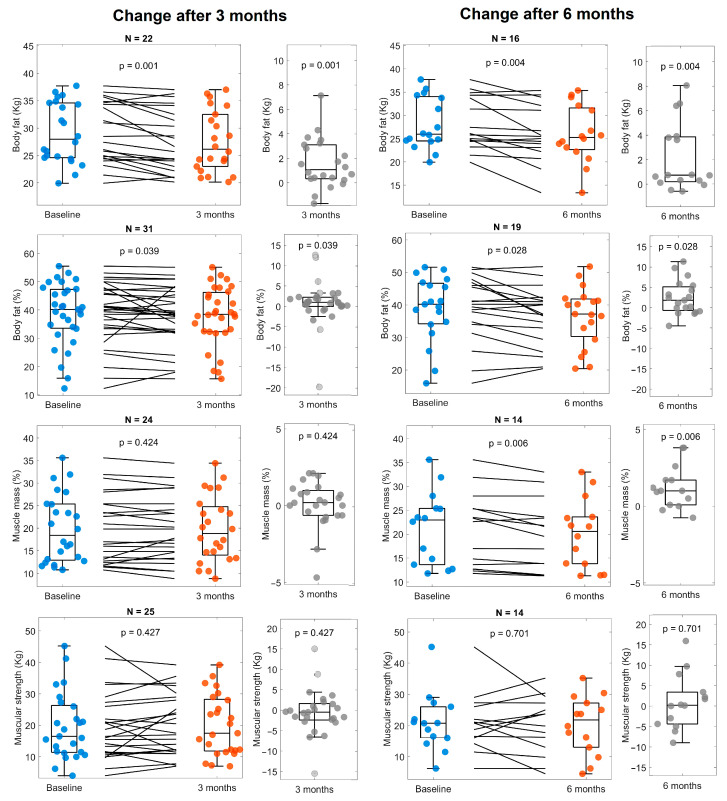
Body composition and arm dynamometry at baseline, 3 and 6 months. The panels on the left compare values at baseline (blue) with 3 months (orange), as well as the change between them (gray). Panels on the right show the changes observed after 6 months (orange) compared with baseline (blue) and the change between them (gray). The number of subjects included for the analysis of every variable is shown at the top of every panel.

**Figure 6:**
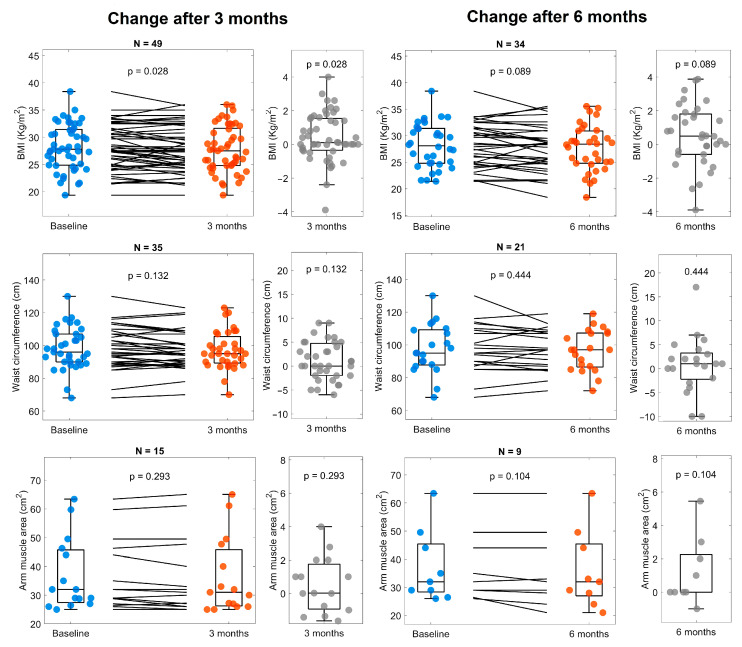
Body mass index (BMI), waist circumference, and arm muscular area at baseline and 3 and 6 months. The panels on the left compare values at baseline (blue) with three months (orange), as well as the change between them (gray). Panels on the right show the changes observed after 6 months (orange) compared with baseline (blue) and the change between them (gray). The number of subjects included for analyzing every variable is shown at the top of every panel.

The patients showed a decrease in fat percentage at 3 months and muscle mass percentage at 6 months (Figure 5). However, body mass index (BMI), waist circumference, and arm muscle area did not change (Figure 6). We did not find statistically significant changes in other parameters of body composition.

Supplementary Material Tables S1–S7 provide the exact values of the presented variables, as well as other biochemical and body composition parameters, such as those obtained from complete blood count and body water distribution.

## 4. Discussion

In this retrospective and observational study, we show the effects of Cetolán III supplementation in a protein-restricted diet in CKD patients. We observed a discrete increase in eGFR and a decrease in creatinine, body fat percentage, and glucose over the follow-up, but we did not observe a change in other biochemical parameters. Furthermore, the percentage of patients presenting with CKD-related symptoms seemed to decrease over time.

To compensate for the protein deficit in the diet, supplementation with ketoanalogues (KAs), which are precursors of essential amino acids through biochemical catalyzing by transaminases, was used [1]. In KAs, the chemical amino group is replaced by a keto group, so theoretically, they reduce the contribution of uremic toxins to the diet without reducing the contribution of essential amino acids [1]. Several efforts have been made to perform randomized controlled trials to test the potential improvement in nutritional status after a low-protein diet and KAs [16]. In adults with CKD pre-dialysis, KA supplementation slowed progression towards renal replacement therapy [17,18]. A very-low-protein diet plus KA supplementation has also been shown to ameliorate mineral bone disorder in CKD by decreasing serum phosphate levels and preventing hyperthyroidism in several clinical trials [19,20,21]. However, other clinical trials did not find a statistically significant difference in the progression of CKD or mineral metabolism parameters [3,17,19,22]. In our observational study, we did not find any difference in electrolytes, including phosphorus levels, but we found that eGFR had a significant increase in patients after KA supplementation. Also, we observed a decrease in uremic toxins (i.e., creatinine and uric acid). Interestingly, the BMI of the patients in our studies decreased slightly at 3 months, but at 6 months, this difference had disappeared. A clinical trial by Garneata et al. did not find a significant change in BMI either [18]. It is important to note that the abovementioned clinical trials combined low- and very-low-protein diets with KA supplementation alone, whereas in our study, the results were obtained by low-protein diets and KA supplementation plus calcium and inulin.

Adults with CKD pre-renal replacement therapy are advised to have a total elemental calcium intake of 800–1000 mg per day to maintain a neutral calcium balance and prevent osteoporosis [7]. Calcium supplementation has been demonstrated to mitigate the progression of CKD and to improve metabolic acidosis and kidney remodeling in animal models [23]. Furthermore, dietary calcium citrate supplementation has a protective effect on nephrons (anatomical and functional units of the kidneys), since it mitigates metabolic acidosis (partially after citrate transformation to bicarbonate in the liver), decreases proteinuria, and improves glomerular filtration rate [24]. A clinical trial that included patients with CKD and secondary hyperparathyroidism showed that phosphorus restriction combined with calcium supplementation mitigates hyperparathyroidism [25]. Another two small crossover studies showed that a high-calcium diet and elemental calcium supplementation resulted in a more favorable calcium balance. However, no clinically significant changes in parathyroid hormone were identified [26,27]. In our work, a detailed description of parathyroid hormone levels was not feasible. However, we did not find any changes in phosphate and calcium levels consistent with the aforementioned studies [26,27].

Little information is available about the effects of inulin supplementation in CKD, and there are no specific recommendations for prebiotic fibers in CKD [28], although it is suggested that they may improve gastrointestinal symptoms (constipation and diarrhea) and aid in calcium absorption [28,29]. A recent clinical trial that included a total of 41 CKD patients on a low-protein diet with and without inulin supplementation for six months showed that those who took inulin had improved clinical and psycho-cognitive assessments [9]. Specifically, the group with inulin showed a decrease in total cholesterol, triglycerides, and higher HDL compared to baseline. However, we did not observe such changes in our retrospective study. The same study [9] also reported an improvement in physical functioning, bodily pain, social functioning, and general health perception. Although we did not measure such variables, we did find a decrease in the severity of CKD symptoms after Cetolán III supplementation.

An animal model has shown that KAs, inulin, and calcium citrate (Cetolán III) have a nephroprotective effect against ischemia–reperfusion damage, as they reduce the levels of biomarkers of kidney damage and inflammation [8]. The damage was caused by ischemia–reperfusion, often used in experimental models to study acute kidney injury. In one such study, Sánchez-Martínez et al. [8] concluded that Cetolán III has nephroprotective effects. Despite the positive findings in our study, given the methodological design, it is not possible to link Cetolán III as the cause of the apparent improvement in eGFR.

### Limitations and Perspectives

Our work has several limitations, from the methodological design to the bias introduced by data loss during follow-up [30]. There are some advantages to using retrospective cohorts to explore new topics, such as in the present study: it is relatively inexpensive, it can be performed almost immediately, and helps to formulate new hypotheses that are useful for future research. However, retrospective cohorts allow us poor control over the exposure factor (Cetolán III), covariates, and potential confounders [31]. Therefore, we cannot be sure that the nutritional supplementation in this study was the cause of eGFR and uremic improvement or the improvement in symptom severity, but rather a correlation among these. Nonetheless, this work shows the potential effects of Cetolán III that should be studied prospectively in a larger sample to obtain robust data for eventual clinical trials to establish the biological effects of Cetolán III supplementation. Furthermore, other research methods, such as a clinical trial, would allow investigation of the effects of other covariables: primary cause of CKD, comorbidities, and other drugs taken by the patients.

One of the future perspectives for this report is to extend the follow-up beyond six months because CKD progresses relatively slowly, especially during the first years of diagnosis [32]. Furthermore, a review of recent scientific literature [33] suggested that various ethnic and cultural differences in diet may be a confounding factor in the clinical effect of KA supplementation. Little is known about the effect of KAs in the Mexican population. Moreover, KA research is of great clinical interest, since delaying renal deterioration in patients with CKD offers an opportunity to reduce the costs of outpatient renal replacement therapy. In Mexico, outpatient renal replacement therapy varies between USD 35 and 150 per session, which dramatically exceeds the income of patients with end-stage ESRD since the monthly individual or family income of these patients is less than USD 110 in 52.3% of them [33]. Importantly, no new symptoms were reported by patients during Cetolán III supplementation. This might indicate good drug tolerability, although more research is needed on this aspect of the therapy [34].

Although creatinine and urea have common metabolic pathways, most of the urea synthesis occurs in the liver, and the primary source is dietary protein. Therefore, the amount of urea produced and circulating in the blood also varies with substrate delivery to the liver and the adequacy of liver function [35]. The latter is highly influenced by catabolic processes, such as fever, infection, and drugs [35]. On the other hand, creatinine is taken in and stored by the muscle tissue as creatine phosphate after a complex series of synthesis processes occurring in the kidneys, mucosa of the small intestine, pancreas, and liver. Contrary to urea, creatinine reflects body mass and is unaffected by catabolic factors [35]. More studies are required to confirm whether the discrepancy in urea and creatinine levels is due to statistical power and study design or caused by the intake of KAs. A decrease in glucose was also observed at 3 and 6 months, with no relevant changes in other laboratory parameters.

Regarding body composition, a decrease in fat mass (at 3 and 6 months) and muscle mass (at six months) was observed, without change in other composition parameters or anthropometry (body mass index, waist circumference, arm muscle area). This result was obtained from only 11 patients whose muscle mass was measured at the 6-month follow-up. Furthermore, the loss of muscle mass is an expected outcome during CKD due to the dysregulation of muscle protein metabolism and impaired muscle cell regeneration [36], which is not shown to improve after Cetolán III supplementation.

## 5. Conclusions

Our study is the first investigation on combined KA, calcium, and inulin supplementation in humans. The results obtained from this retrospective cohort point to a correlation between improvement in the severity of CKD-related symptoms, body composition, and eGFR by combined supplementation with KAs, inulin, and calcium citrate. However, due to our methodological limitations, future studies are required to establish causal relationships.

## Figures and Tables

**Table 1:** Demographic and clinical characteristics of patients included in this study. Data reported as medians (percentile 25–percentile 75).

Variable	N = 76
Age (years)	71 (64–77)
Sex	
Female	43 (57%)
Males	33 (43%)
Time since CKD diagnosis (months)	12 (3–24)
DM	
Time since DM diagnosis (months)	240 (48–276)
HTN	65 (86%)
Time since HTN diagnosis (months)	180 (48–240)
Autoimmune disease	1 (1%)
Edema	2 (3%)
Smoking	2 (3%)
Alcoholism	32 (44%)
Drug consumption	5 (7%)

CKD: chronic kidney disease; DM: diabetes mellitus; HTN: hypertension.

## Data Availability

The raw data supporting this article’s conclusions will be made available upon request to the corresponding author, provided pertinent legal requirements are met.

## Supplementary Materials

The following supporting information can be downloaded at https://www.mdpi.com/article/10.3390/life14121638/s1. Table S1: Uremic toxins and eGFR at baseline and after 3 and 6 months of dietary supplementation with Cetolán III. Data are shown as medians (percentile 25–percentile 75); Table S2: Glucose, total cholesterol, triglycerides, LDL, and HDL at baseline and after 3 and 6 months of dietary supplementation with Cetolán III. Data are shown as medians (percentile 25–percentile 75); Table S3: Serum albumin, hematocrit, hemoglobin, and iron at baseline and after 3 and 6 months of dietary supplementation with Cetolán III. Data are shown as medians (percentile 25–percentile 75); Table S4: Serum electrolytes at baseline and after 3 and 6 months of dietary supplementation with Cetolán III. Data are shown as medians (percentile 25–percentile 75); Table S5: Body composition and arm dynamometry at baseline and after 3 and 6 months of dietary supplementation with Cetolán III. Data are shown as medians (percentile 25–percentile 75); Table S6: Total water, extracellular water, and phase angle evaluated at baseline and after 3 and 6 months of dietary supplementation with Cetolán III. Data are shown as medians (percentile 25–percentile 75); Table S7: Body mass index (BMI), waist circumference, and muscular area of the arm at baseline and after 3 and 6 months of dietary supplementation with Cetolán III. Data are shown as medians (percentile 25–percentile 75).
