# Supplementary material for: Diet Supplementation with Ketoanalogues, Inulin, and Calcium Citrate in Chronic Kidney Disease: A Retrospective Cohort
Source: Life (Basel). 2024 Dec 10;14(12):1638. doi: 10.3390/life14121638 (PMC11677553; doi:10.3390/life14121638)
Supplement: Supplementary file 1 [file life-14-01638-s001.zip › life-3228216-supplementary.pdf]

Supplementary material

TableS1. Uremic toxins and eGFR at baseline, and 3 and 6 months of dietary supplementation with Cetolán III. Data is shown as median (percentile 25 – percentile 75).

	Change after 3 months				Change after 6 months			
**Variable**	**Baseline**	**3 months**	**Δ 3 months**	**N**	**Baseline**	**6 months**	**Δ 6 months**	**N**
Urea (mg/dL)	70.6 (45.8 – 121.0)	61.4 (44.0 – 102.0)	-11.9 (-20.0 – 10.0)	34	70.0 (40.0 – 110.3)	69.6 (42.0 – 101.0)	-4.3 (-12.6 – 9.0)	25
eGFR (mL/min/1.73m^2)	27.0 (17.0 – 44.0)	25.3* (14.0 – 45.0)	1.9 (-2.0 – 5.1)	54	29.0 (18.0 – 45.0)	30.0* (22.0 – 47.0)	3.0# (0.0 – 10.0)	37
Creatinine (mg/dL)	1.69 (1.26 – 2.67)	1.84* (1.26 – 3.06)	-0.11 (-0.32 – 0.07)	40	1.69 (1.22 – 3.00)	1.60* (1.20 – 2.78)	-0.17# (-0.43 – 0.11)	30
Uric acid (mg/dL)	7.50 (6.13 – 9.15)	6.90 (5.05 – 8.50)	-0.8 (-1.9 – 0.8)	40	7.25 (5.80 – 8.80)	6.30 (5.04 – 7.40)	-0.5# (-2.5 – 0.2)	26

eGFR = estimated glomerular filtration rate

* $p < 0.05$ versus baseline (Wilcoxon's signed rank test)

# $p < 0.05$ versus a Δ value different from zero (One-sample signed rank test)

Table S2. Glucose, total cholesterol, triglycerides, LDL and HDL at baseline, 3, and 6 months of dietary supplementation with Cetolán III. Data is shown as median (percentile 25 – percentile 75).

	**Change after 3 months**				**Change after 6 months**			
**Variable**	**Baseline**	**3 months**	**Δ 3 months**	**N**	**Baseline**	**6 months**	**Δ 6 months**	**N**
Glucose (mg/dL)	105.9 (91.0 – 134.0)	94.8* (81.0 – 105.0)	-7.5# (-22.2 – 7.1)	46	106.7 (92.0 – 134.0)	97.0 (83.0 – 116.0)	-8.0# (-35.0 – 5.0)	31
Total cholesterol (mg/dL)	151.0 (131.0 – 172.0)	158.0 (139.0 – 131.0)	1.0 (-10.0 – 24.0)	33	150.0 (141.0 – 160.0)	155.9 (121.0 – 178.0)	-7.2 (-19.6 – 13.0)	19
Triglycerides (mg/dL)	139.0 (98.0 – 164.0)	123.4 (107.6 – 163.0)	2.0 (-20.0 – 35.0)	27	126.0 (100.3 – 147.0)	119.5 (109.0 – 144.4)	-5.0 (-22.0 – 7.9)	17
LDL (mg/dL)	100.0 (76.4 – 115.9)	98.3 (76.0 – 119.0)	-6.0 (-25.0 – 14.4)	25	76.2 (67.7 – 97.0)	85.8 (53.9 – 94.0)	-0.5 (-12.0 – 7.3)	10
HDL (mg/dL)	43.0 (35.0 – 49.0)	49.0 (39.1 – 55.0)	4.0# (-1.0 – 10.0)	23	48.0 (32.2 – 49.0)	45.0 (29.7 – 51.0)	-1.2 (-4.6 – 1.0)	11

LDL = low density lipids; HDL = high density lipids.

* $p < 0.05$ versus baseline (Wilcoxon's signed rank test)

# $p < 0.05$ versus a Δ value different from zero (One-sample signed rank test)

Table S3. Serum albumin, hematocrite, hemoglobin and iron at baseline, and 3 and 6 months of dietary supplementation with Cetolán III. Data is shown as median (percentile 25 – percentile 75).

	Change after 3 months				Change after 6 months			
**Variable**	**Baseline**	**3 months**	**Δ 3 months**	**N**	**Baseline**	**6 months**	**Δ 6 months**	**N**
Albumin (g/dL)	4.00 (3.96 – 4.20)	4.20 (3.60 – 4.41)	0.10 (-0.20 – 0.40)	21	4.00 (3.95 – 4.20)	4.00 (3.60 – 4.44)	-0.10 (-0.35 – 0.32)	14
Hematocrite (%)	36.1 (30.4 – 40.9)	38.0 (32.6 – 39.4)	0.9 (-2.6 – 4.1)	26	34.2 (30.4 – 39.0)	35.0* (34.4 – 43.7)	1.9# (0.2 – 6.7)	15
Hemoglobine (g/dL)	11.3 (9.2 – 13.3)	12.4 (10.7 – 13.5)	0.3 (-0.7 – 1.5)	26	10.7 (9.2 – 11.9)	12.0* (10.9 – 14.0)	1.5# (0.2 – 1.9)	15
Iron (mcg/dL)	85.0 (62.0 – 91.5)	69.0 (53.0 – 92.0)	-5.1 (-32.0 – 13.0)	11	85.0 (44.1 – 91.5)	76.6 (48.0 – 100.0)	-3.0 (-14.0 – 35.9)	7

* $p < 0.05$ versus baseline (Wilcoxon's signed rank test)

# $p < 0.05$ versus a Δ value different from zero (One-sample signed rank test)

Table S4. Serum electrolytes, and 3 and 6 months of dietary supplementation with Cetolán III. Data is shown as median (percentile 25 – percentile 75).

	Change after 3 months				Change after 6 months			
**Variable**	**Baseline**	**3 months**	**Δ 3 months**	**N**	**Baseline**	**6 months**	**Δ 6 months**	**N**
Calcium (mg/dL)	9.40 (8.80 – 9.70)	9.00 (8.80 – 9.60)	0.00 (-0.20 – 0.30)	26	9.40 (9.10 – 9.80)	9.20 (9.00 – 9.70)	-0.10 (-0.30 – 0.00)	13
Sodium (mEq/L)	138.3 (136.0 – 140.0)	137.1 (136.0 – 141.0)	0.0 (-1.0 – 2.0)	26	138.5 (136.0 – 141.1)	139.0 (138.0 – 140.0)	0.0 (-3.0 – 2.0)	15
Phosporus (mEq/L)	3.85 (3.30 – 4.49)	3.70 (3.52 – 4.40)	-0.10 (-0.50 – 0.40)	22	3.45 (3.30 – 3.70)	3.60 (3.30 – 3.90)	0.10 (0.00 – 0.20)	10
Potasium (mEq/L)	4.98 (4.30 – 5.28)	4.75 (4.50 – 5.10)	0.03 (-0.30 – 0.40)	22	4.89 (4.36 – 5.29)	4.60 (4.10 – 5.00)	-0.35 (-0.60 – 0.23)	12

* $p < 0.05$ versus baseline (Wilcoxon's signed rank test)

# $p < 0.05$ versus a Δ value different from zero (One-sample signed rank test)

Table S5. Body composition and arm dynamometry at baseline, and 3 and 6 months of dietary supplementation with Cetolán III. Data is shown as median (percentile 25 – percentile 75).

	**Change after 3 months**				**Change after 6 months**			
**Variable**	**Baseline**	**3 months**	**Δ 3 months**	**N**	**Baseline**	**6 months**	**Δ 6 months**	**N**
Fat mass (Kg)	28.0 (24.6 – 34.6)	26.2* (23.0 – 32.5)	-1.1# (-3.1 – -0.3)	22	26.0 (24.5 – 34.0)	25.2* (22.7 – 31.6)	-0.7# (-3.8 – -0.2)	16
Fat mass (%)	40.2 (33.4 – 47.4)	38.5* (32.3 – 46.5)	-1.0# (-2.3 – 0.0)	31	40.2 (34.0 – 46.9)	37.2 (29.4 – 42.0)	-1.8# (-5.4 – 0.9)	19
Muscle mass (%)	18.4 (12.9 – 25.4)	18.8 (14.0 – 24.7)	-0.2 (-1.0 – 0.6)	24	23.0 (13.6 – 25.4)	20.6* (13.8 – 23.6)	-1.0# (-1.7 – -0.1)	14
Muscular strength (%)	16.5 (11.5 – 26.0)	17.5 (12.0 – 28.0)	0.5 (-1.5 – 2.3)	25	20.8 (16.0 – 26.0)	21.8 (13.0 – 27.2)	-0.2 (-3.4 – 4.4)	14

* $p < 0.05$ versus baseline (Wilcoxon's signed rank test)

# $p < 0.05$ versus a Δ value different from zero (One-sample signed rank test)

Table S6. Total water, extracellular water, and phase angle were evaluated at baseline, 3 and 6 months of dietary supplementation with Cetolán III. Data is shown as median (percentile 25 – percentile 75).

	Change after 3 months				Change after 6 months			
**Variable**	**Baseline**	**3 months**	**Δ 3 months**	**N**	**Baseline**	**6 months**	**Δ 6 months**	**N**
Total water (L)	28.3 (25.7 – 36.0)	29.6 (25.6 – 35.4)	-0.1 (-0.7 – 0.7)	27	28.5 (27.0 – 36.0)	28.6 (25.0 – 38.1)	-0.8 (-1.1 – 0.3)	18
Extracellular water (L)	14.3 (13.6 – 17.0)	14.4 (13.4 – 17.1)	0.1 (-0.6 – 0.7)	27	14.8 (13.6 – 16.4)	15.5 (13.1 – 17.9)	0.1 (-0.5 – 0.9)	18
Phase angle (°)	4.20 (4.00 – 4.50)	4.20 (3.90 – 4.10)	-0.20 (-0.30 – 0.10)	15	4.20 (4.00 – 4.67)	4.10 (3.80 – 4.40)	-0.10 (-0.40 – 0.10)	11

* $p < 0.05$ versus baseline (Wilcoxon's signed rank test)

# $p < 0.05$ versus a Δ value different from zero (One-sample signed rank test)

Table S7. Body mass index (BMI), waist circumference, and muscular area of the arm at baseline, 3 and 6 months of dietary supplementation with Cetolán III. Data is shown as median (percentile 25 – percentile 75).

	Cambio a 3 meses de tratamiento				Cambio a 6 meses de tratamiento			
**Variable**	**Basal**	**3 meses**	**Δ 3 meses**	**N**	**Basal**	**6 meses**	**Δ 6 meses**	**N**
BMI (Kg/m^2)	27.8 (24.9 – 31.4)	27.5 (24.8 – 31.5)	-0.1# (-1.5 – 0.3)	49	28.1 (24.8 – 31.4)	28.4 (24.8 – 30.9)	-0.5 (-1.8 – 0.6)	34
Waist circumference (cm)	96.0 (90.0 – 107.0)	95.0 (89.0 – 107.0)	0.0 (-5.0 – 2.0)	37	95.0 (88.0 – 109.0)	97.0 (87.0 – 107.0)	-1.0 (-3.0 – 2.0)	21
Muscular area of the arm (cm^2)	32.0 (27.0 – 46.3)	31.0 (26.0 – 47.7)	0.0 (-2.0 – 1.0)	15	32.0 (29.0 – 44.0)	32.0 (28.0 – 44.0)	0.0 (-2.0 – 0.0)	9
Energy intake (%)	75.3 (60.0 – 101.0)	80.0 (70.0 – 92.0)	5.0 (-23.8 – 18.0)	50	85.0 (65.0 – 110.0)	82.2 (70.0 – 90.0)	2.8 (-31.5 – 20.0)	43

* $p < 0.05$ versus baseline (Wilcoxon's signed rank test)

# $p < 0.05$ versus a Δ value different from zero (One-sample signed rank test)
